# High-resolution US of the facial vessels with new facial vein landmarks for reconstructive surgery and dermal injection

**DOI:** 10.1186/s41747-023-00363-8

**Published:** 2023-09-11

**Authors:** Federico Pistoia, Paola Lovino Camerino, Alessandro Ioppi, Riccardo Picasso, Federico Zaottini, Simone Caprioli, Davide Mocellin, Alessandro Ascoli, Michelle Pansecchi, Andrea Luigi Camillo Carobbio, Giampiero Parrinello, Filippo Marchi, Giorgio Peretti, Carlo Martinoli

**Affiliations:** 1 IRCCS Ospedale Policlinico San Martino, Largo Rosanna Benzi, 10, Genoa, Italy; 2 Department of Surgical Sciences and Integrated Diagnostics (DISC), University of Genova, Genoa, Italy; 3 Department of Otorhinolaryngology, Ospedale S. Paolo, Savona, Italy; 4 Department of Otorhinolaryngology, Ospedale Giovanni Borea, Sanremo, Italy; 5 Department of Health Sciences (DISSAL), Radiology Section, University of Genova, Via Pastore 1, Genoa, Italy; 6 Department of Neurosciences, Section of Otorhinolaryngology—Head and Neck Surgery, University of Padua “Azienda Ospedaliera Di Padova”, 35128 Padua, Italy; 7 Department of Internal Medicine (DIMI), University of Genoa, Genoa, Italy

**Keywords:** Arteries, Face, Reconstructive surgical procedures, Ultrasonography, Veins

## Abstract

**Background:**

Accurate knowledge of vessel anatomy is essential in facial reconstructive surgery. The technological advances of ultrasound (US) equipment with the introduction of new high-resolution probes improved the evaluation of facial anatomical structures. Our study had these objectives: the primary objective was to identify new surgical landmarks for the facial vein and to verify their precision with US, the secondary objective was to evaluate the potential of high-resolution US examination in the study of both the facial artery and vein.

**Methods:**

Two radiologists examined a prospective series of adult volunteers with a 22–8 MHz hockey-stick probe. Two predictive lines of the facial artery and vein with respective measurement points were defined. The distance between the facial vein and its predictive line (named mandibular-orbital line) was determined at each measurement point. The distance from the skin and the area of the two vessels were assessed at every established measurement point.

**Results:**

Forty-one volunteers were examined. The median distance of the facial vein from its predictive line did not exceed 2 mm. The facial vein was visible at every measurement point in all volunteers on the right side, and in 40 volunteers on the left. The facial artery was visible at every measurement point in all volunteers on the right and in 37 volunteers on the left.

**Conclusions:**

The facial vein demonstrated a constant course concerning the mandibular-orbital line, which seems a promising clinical and imaging-based method for its identification. High-resolution US is valuable in studying the facial artery and vein.

**Relevance statement:**

High-resolution US is valuable for examining facial vessels and can be a useful tool for pre-operative assessment, especially when combined with the mandibular-orbital line, a new promising imaging and clinical technique to identify the facial vein.

**Key points:**

• High-resolution US is valuable in studying the facial artery and vein.

• The facial vein demonstrated a constant course concerning its predictive mandibular-orbital line.

• The clinical application of the mandibular-orbital line could help reduce facial surgical and cosmetic procedure complications.

**Graphical Abstract:**

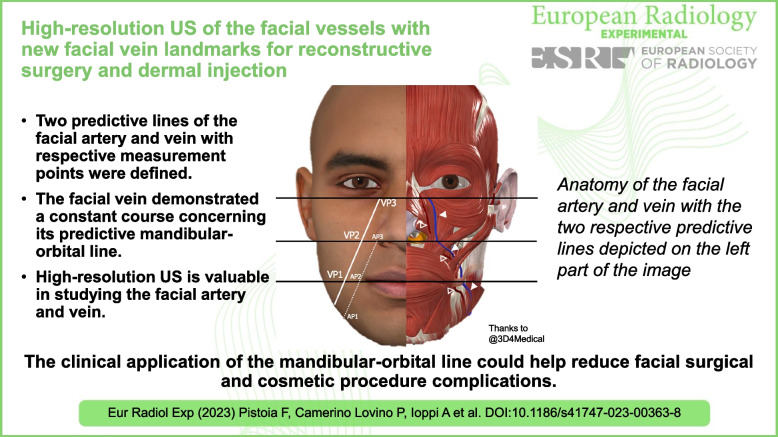

## Background

The understanding of facial vessel anatomy is essential for reconstructive, plastic, and aesthetic procedures concerning the head and neck district. Precisely, the harvesting of flaps for the reconstruction of the oral cavity, the lips, the eyelids, or nasal subunits requires an accurate knowledge of the course of these vessels, and frequently a preoperative identification by noninvasive methods [1, 2]. Indeed, for pedicled flaps such as the facial artery myocutaneous flap (FAMM) and the islanded nasolabial flap, particular attention must be paid to studying and safeguarding their vascular supply [3, 4]. Moreover, in free flap reconstruction of the upper and midface, the distal tract of the facial pedicle is the ideal recipient when the length of the flap pedicle is not enough to reach the neck. Aesthetic procedures performed in the facial district are mostly represented by dermal filler and neurotoxin injection [5]. Since the most feared complications of such procedures are direct vascular injury and the vascular obstruction caused by the material injected (mostly hyaluronic acid) [5–8], the surgeon who performs these procedures should be aware of the detailed vascular anatomy of the facial vessels [9]. Therefore, the course of the facial artery and its branches has been long studied to improve its clinical identification [10–12]. On the contrary, only a few studies have focused on the anatomy of the facial veins [13, 14].

Nowadays, the preoperative identification of the facial artery is performed either by direct palpation, perceiving its pulsation on the mandibular rim, or with the aid of Doppler probes, which can help in identifying the artery even distally, in the proximity of the cheilion or the nasal ala. Notably, the identification of the facial vein remains an issue, since these methods are not able to detect it. Moreover, continuous-wave Doppler are incapable of measuring the diameter and depth of the vessels. In addition, patient-related features such as excessive subcutaneous tissue, hypotension, and previous irradiation/surgical procedures of the head and neck region can burden the correct vessel detection.

In previous studies, color Doppler US was able to detect the facial artery and vein at the lower border of the mandible, around the oral commissure, and under the nasal ala [15, 16]. Furthermore, the recent introduction of high-resolution US probes, specifically designed for small parts examinations, has significantly improved the spatial and contrast resolution of the image, refining the evaluation of the anatomical structures of the face and thus opening new perspectives in the study of the facial artery and vein [17, 18].

Therefore, our study had these objectives: the primary objective was to identify new surgical landmarks for the facial vein and to verify the precision of these landmarks with US, the secondary objective was to evaluate the applicability of high-resolution US examination in the study of both the facial vein and artery.

## Methods

### Subjects and US protocol

This study was approved by the local Ethics Committee (Registry number CER Liguria: 128,269) and informed consent was obtained from all volunteers. Following a training to adopt the same sonographic technique, two experienced sonologists, with specific skills in musculoskeletal and vascular imaging, examined a consecutive series of adult volunteers enrolled between August 2021 and May 2022. Each sonologist examined a different group of volunteers. During the US examination, a large amount of gel was applied between the probe and the skin of the patients in order to avoid any alteration of the results due to soft tissue compression. The US machine was equipped with a high-frequency, 8-mm footprint, 22–8 MHz hockey-stick transducer (Aplio i800, Canon Medical System, Ōtawara, Japan).

Inclusion criteria were the absence of any history of pathology or surgical procedures on the face. Patients who underwent neck surgeries that may have conditioned the vascular anatomy of the facial vessels were excluded.

**Fig. 1:**
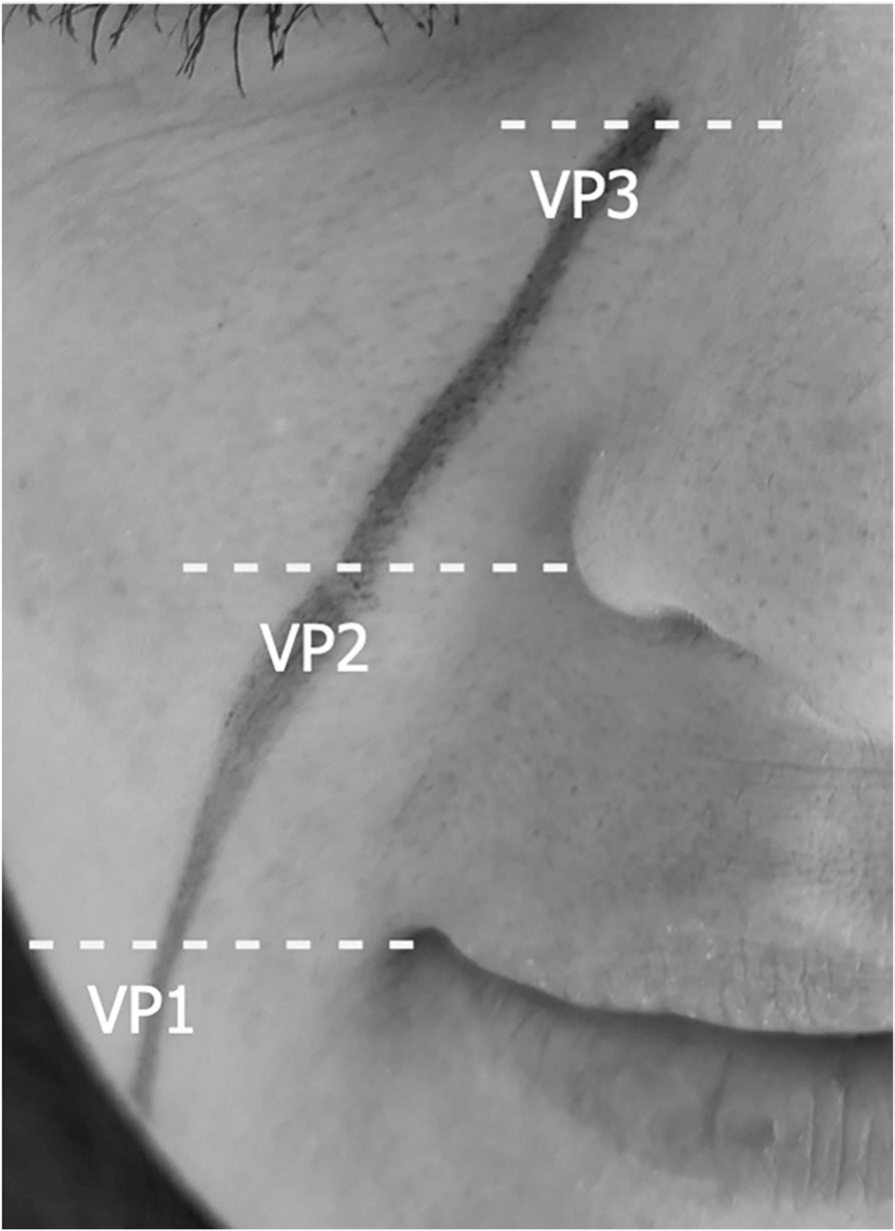
The MO-line (black drawn line) with the levels (dotted lines) of the three venous measurement points (VP1, 2, and 3). *VP* Venous point

**Fig. 2:**
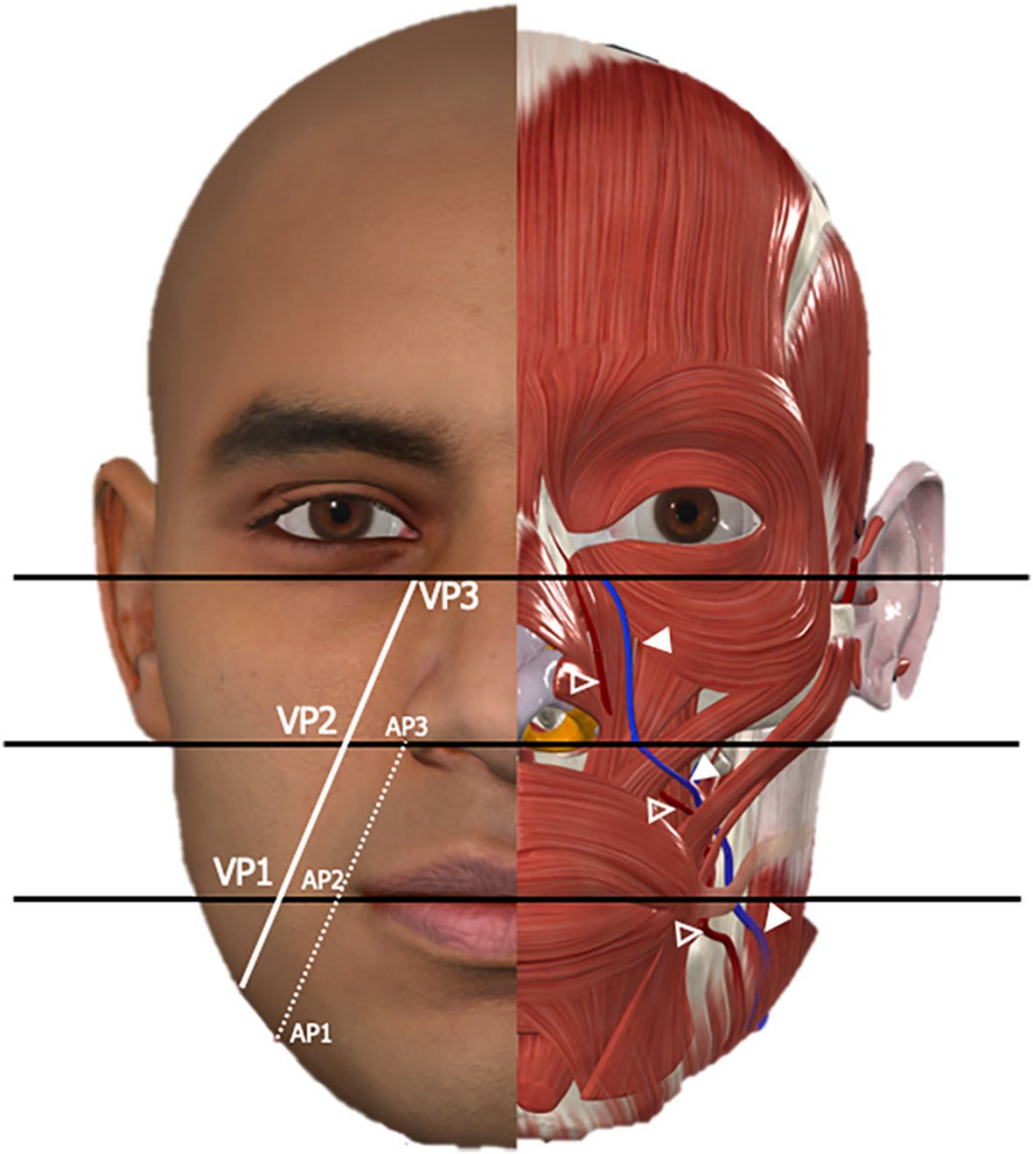
Facial artery and vein anatomy. The facial vein (white arrowhead, depicted in blue) originates at the lower margin of the orbit and courses obliquely down the face to the angle of the mandible. In the infraorbital region the facial vein travels between the *orbicularis oculi* muscle and the *levator labii superioris alaeque nasi* muscle; caudally, the facial vein is located deep to the *zygomaticus major muscle* and to the *risorius* muscle. The facial artery (void arrowhead, depicted in red) travels obliquely along the antero-inferior border of the *masseter *muscle towards the cheilion, passes deep to the *risorius* and *zygomaticus major* muscles and variably over or under the *levator labii superioris* muscle to terminate as angular artery at the medial commissure of the eye. On the right part of the image, the mandibular-orbital line (white), with the levels (black lines) of the three venous measurement points (VP1, 2, and 3), and the three arterial measurement points (AP1, 2, and 3) along the line (dotted line) drawn from the facial artery at the lower border of the mandible toward the inferolateral angle of the nasal ala. *VP* Venous point, *AP* Arterial point. Image courtesy of Complete Anatomy

The facial artery and vein of each side were evaluated as follows. In each participant, the probe was placed at the lower border of the mandible over the facial artery previously identified by palpation. Once the facial artery and the facial vein in its proximity were visualized in the transverse axis, the digital calipers of the machine were used to measure the distance between the two vessels, together with the vein area and its depth beneath the skin. Then, using an elastic ruler, a straight line following the presumed course of the facial vein was drawn on the skin of the subject from the facial vein at the angle of the mandible to the medial canthus. This line was called the mandibular-orbital line (MO-line). Along the MO-line, three measurement points were arbitrarily established:

- The venous point 1 (VP1) at the intersection of the MO-line with a virtual horizontal line running through the cheilion;
- The venous point 2 (VP2) at the intersection of the MO-line with a virtual horizontal line passing through the nasal ala;
- And the venous point 3 (VP3) at the intersection between the MO-line and the medial infra-orbital margin (Figs. 1 and 2).

The digital calipers of the US machine were used to measure the distance between each measurement point (VP1, VP2, and VP3) and the course of the facial vein, after aligning the center of the probe (as indicated by the centerline marks of the transducer) to the MO-line. Once the measurement button was pushed, the digital calipers were automatically placed at the center of the image by the US machine. In addition, at each point, the vein area and the depth beneath the skin were recorded. An analogous straight line was drawn from the facial artery at the lower border of the mandible toward the inferolateral angle of the nasal ala (Fig. 2). The facial artery was studied with US from the lower border of the mandible to the nasal ala. Three measurement points were arbitrarily established: the arterial point 1 (AP1) at the lower border of the mandible, the arterial point 2 (AP2) at the level of a virtual horizontal line running through the cheilion, and the arterial point 3 (AP3) at the nasal alar base. In every AP were measured the facial artery section area and its depth beneath the skin. During the US examination, the facial vein was distinguished from the artery thanks to its compressibility and the blood flow on Doppler.

### Statistical analysis

As this is an observational non-comparative study, no prespecified sample size calculation was needed. We enrolled a consecutive series of volunteers willing to participate in the study. Mean, median, standard deviation and ranges were calculated for each of the reported values where appropriate. Descriptive statistics used numbers and percentages for qualitative variables and median and interquartile range (IQR) for quantitative variables. Differences between groups were compared using the chi-square test or the Fisher’s exact test where appropriate; *p* values < 0.05 were considered statistically significant. All statistical calculations were performed using SPSS v25.

## Results

A consecutive series of 41 Caucasian adult volunteers, 21 females (51.2%), 39 right-handed (95.1%) ranging in age from 26 to 61 years (32 ± 11) was examined.

**Fig. 3:**
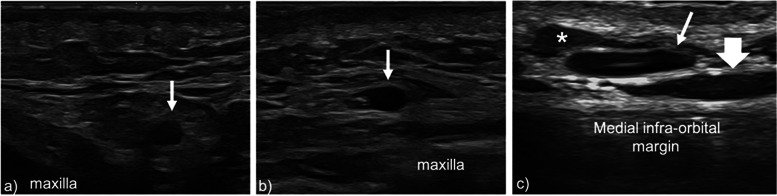
Transverse 22–8 MHz ultrasound images show the facial vein running in the subcutaneous tissue at VP1 (**a**) and VP2 (**b**). **c** Transverse 22–8 MHz US image at VP3 demonstrates the facial vein (white arrow) between the *orbicularis oculi* muscle (asterisk) and the *levator labii superioris* muscle (large arrow). *VP* Venous point

**Fig. 4:**
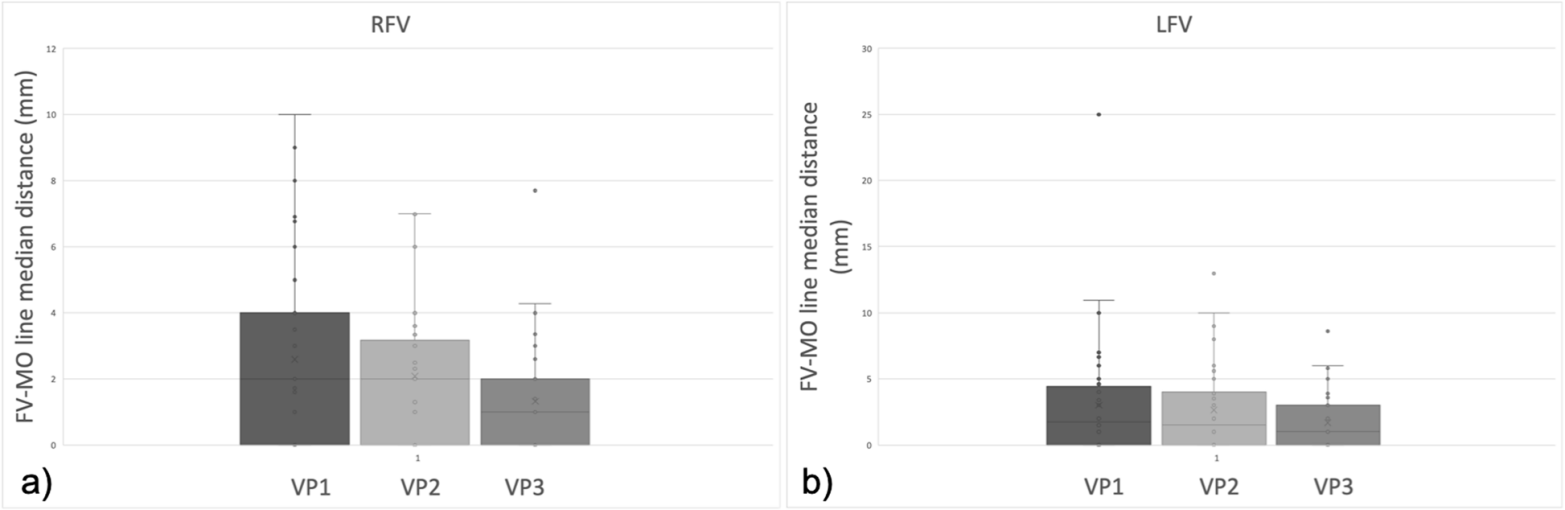
Median distance between the facial vein and the MO-line at the VP1, 2, and 3 on the right (**a**) and the left (**b**) side. Bars indicate the interquartile range and the horizontal line through the box shows the median. Dots represent outliers. *FV* Facial vein, *MO-line* Mandibular-orbital line, *VP* Venous point, *RFV* Right facial vein, *LFV* Left facial vein

High-resolution US was able to visualize the facial vein at the lower border of the mandible and at VP1, VP2, and VP3 in 41/41 (100%) volunteers on the right side and in 40/41 (97.6%) volunteers on the left side (Fig. 3), without significant difference between the two sides (*p* = 1.000). At the lower border of the mandible, the facial vein was located lateral to the facial artery in 41/41 cases (100%) on the right side, and in 38/40 cases (95%) on the left side, without significant difference (*p* = 0.241). Data relative to the facial vein at the lower border of the mandible are summarized in Table 1. The position of the facial vein relative to the MO-line at every measurement point is summarized in Table 2. At VP1, the median distance of the facial vein from the MO-line was 2.0 mm (IQR 4.0 mm) on the right side, and 1.7 mm (IQR 4.3 mm) on the left side; at VP2, it was 2.0 mm (IQR 3.2) on the right side and 1.5 mm (IQR 4.0) on the left side; at VP3, it was 1.0 mm (IQR 2 mm) on the right side and 1.0 mm (IQR 3 mm) on the left side. Tables 3 and 4 and Fig. 4 summarize these results. No significant differences between the right and left sides were found.

**Table 1:** Mean area and mean depth of the right and left facial vein at the lower border of the mandible with the median distances from the facial artery

FV at lower border of the mandible	Mean area in mm^2^ (SD)	Mean depth beneath skin in mm (SD)	Median distance from the FA in mm (IQR)	*N*
RFV	3.5 (2.8)	8.2 (1.5)	4.7 (2.1–9)	41
LFV	2.7 (1.4)	7.8 (1.6)	3.4 (2–7)	40

*FV* Facial vein*, RFV* Right facial vein*, LFV* Left facial vein*, FA* Facial artery*, N* Number, *SD* Standard deviation*, IQR* Interquartile range

**Table 2:** Position of the FV relative to the MO-line in every measurement point

FV position relative to the MO-line	Lateral *N* (%)	Coincident *N* (%)	Medial *N* (%)	*p* value
RVP1 LVP1	19/41 (46.3) 19/40 (47.5)	16/41 (39) 17/40 (42.5)	6/41 (14.6) 4/40 (10)	0.863
RVP2 LVP2	14/41 (34.1) 9/40 (22.5)	16/41 (39) 17/40 (42.5)	11/41 (26.8) 14/40 (35)	0.481
RVP3 LVP3	6/41 (14.6) 7/40 (17.5)	21/41 (51.2) 18/40 (45)	14/41 (34.1) 15/40 (37.5)	0.848

No significant differences between the right and left side were found (in every point *p* > 0.05). *FV* Facial vein, *LVP* Left venous point, *MO-line* Mandibular-orbital line, *N* Number, *RVP* Right venous point

**Table 3:** Mean area and mean depth of the right facial vein at the VP1, 2, and 3 with the median distances from the MO-line

RFV	Mean area in mm^2^ (SD)	Mean depth beneath skin in mm (SD)	Median distance from the MO-line in mm (IQR)	*N*
VP1	3.7 (2.1)	10.9 (2)	2 (0–4)	41
VP2	3.4 (2.1)	9.1 (2.4)	2 (0–3.2)	41
VP3	4.2 (1.9)	4.9 (6.3)	1 (0–2)	41

*RFV* Right facial vein, *VP* Venous point, *MO-line* Mandibular-orbital line, *N* Number, *SD* Standard deviation, *IQR* Interquartile range

**Table 4:** Mean area and mean depth of the left facial vein at the VP1, 2, and 3 with the median distances from the MO-line

LFV	Mean area in mm^2^ (SD)	Mean depth beneath skin mm (SD)	Median distance from the MO-line mm (IQR)	*N*
VP1	3.9 (1.7)	9.7 (2)	1.7 (0–4.4)	40
VP2	4.3 (2.5)	10.1 (2.5)	1.5 (0–4)	40
VP3	4.8 (2.1)	4.3 (1.5)	1 (0–3)	40

*LFV* Left facial vein*, VP* Venous point*, MO-line* Mandibular-orbital line, *Mm* Millimeters,* N* Number, *SD* Standard deviation*, IQR* Interquartile range

**Fig. 5:**
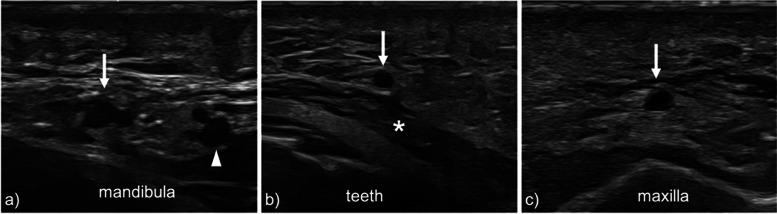
**a** Transverse 22–8 MHz US image shows the facial artery (white arrow) and the adjacent facial vein (arrowhead) at the lower border of the mandible (AP1). **b** Transverse 22–8 MHz US image shows the facial artery (white arrow) running in the subcutaneous tissue superficially to the *buccinator* muscle (asterisk) at AP2. **c** Transverse 22–8 MHz US image demonstrates the facial artery in the subcutaneous tissue below the nasal alar base (AP3), superficially to the maxilla. *AP* Arterial point

On the right side, the facial artery was visible in all 41 volunteers (100%) at AP1, AP2, and AP3; on the left side, the facial artery was visible in all 41 volunteers (100%) at AP1, in 39 volunteers (95.1%) at AP2, and in 37 volunteers (90.2%) at AP3 (Figs. 4 and 5), not significantly different (*p* = 0.949). Data relative to the facial artery are summarized in Tables 5 and 6.

**Table 5:** Mean area and mean depth of the right facial artery at the AP1, AP2, and AP3

RFA	Mean area in mm^2^ (SD)	Mean depth mm (SD)	*N*
AP1	2.2 (1)	6.8 (1.8)	41
AP2	1.6 (0.6)	6.1 (1.5)	41
AP3	1.1 (0.5)	4.3 (0.9)	41

*RFA* Right facial artery*, AP* Arterial point*, N* Number, *SD* Standard deviation

**Table 6:** Mean area and mean depth of the left facial artery at the AP1, AP2, and AP3

LFA	Mean area in mm^2^ (SD)	Mean depth in mm (SD)	*N*
AP1	2.2 (1.1)	6.4 (1.8)	41
AP2	1.7 (0.9)	6.3 (1.4)	39
AP3	1.2 (0.6)	4.6 (1.2)	37

*LFA* Left facial artery*, AP* Arterial point, *N* Number, *SD* Standard deviation

## Discussion

At present, several studies have investigated the course of the facial artery, while only a few focused on the route of the facial vein [10−14]. The number of sonographic studies of the facial vein is even less [15, 16]. The main advantage of US lies in performing a noninvasive *in vivo* examination without the artifacts observed in cadaveric dissections, such as tissue dehydration and vessel collapse, potentially ensuring better reliability of the data collected. The primary disadvantage of US is that this technique is operator-dependent, the main sources of error in US examinations being related to low operator skill, experience, and training. In order to mitigate these factors, the two examiners of our study were experienced sonologists with specific skills in musculoskeletal and vascular imaging and they both trained before the study to standardize their scanning technique. Furthermore, to reduce the operator-dependency of the procedure, the predictive lines were always drawn between the same anatomical landmarks (namely the mandibula border, the medial canthus, and the nasal ala). Regarding the mutual position of the facial artery and vein at the lower border of the mandible and the US visualization of the two vessels in the different measurement points, we did not observe significant differences between the left and the right side. However, given the small sample size of our study, further prospective studies on a wider population are needed to investigate differences between the two sides.

The facial vein originates at the lower margin of the orbit as a continuation of the angular vein and courses obliquely down the face to the angle of the mandible, where it joins the anterior branch of the retromandibular vein to form the common facial vein. The facial artery is one of the branches of the external carotid artery and arises from the carotid triangle. On the face, the facial artery travels obliquely along the antero-inferior border of the masseter muscle towards the cheilion and gives rise to four main vessels: the inferior and superior labial artery, the lateral nasal artery, and the angular artery (the terminal segment of the facial artery). According to Nagase et al. who studied the positional relationship of the facial artery and vein using color Doppler US [16], the two vessels are located together at the lower border of the mandible. This evidence is confirmed by our study, where the mean distance between the facial artery and vein at the lower border of the mandible was 5.6 mm on the right side and 4.4 mm on the left side.

More cranially, the facial artery and vein diverge, as demonstrated by Nagase et al. [16] and supported by the distinct measurement points of the artery and vein used in our study. Koh et al. [19], analyzing 47 Korean specimens, classified the facial artery’s branching pattern into six categories: forehead (4%), angular (36%), nasal (44%), alar (3%), superior labial (7%), and inferior labial (6%). In the perioral area, the facial artery gives off its distal branches, which are collectively known as the perioral arteries that supply the perioral region [20]. Previous anatomical studies on cadavers have demonstrated a horizontal distance of the facial artery from the cheilion between 13.5 and 16 mm, and from the nasal ala of 12 mm [19, 21–23]. In our study, the right facial artery has been correctly identified around the cheilion (AP2) and the nasal ala (AP3) in all 41 (100%) cases, while the left facial artery was visible in 39 (95.1%) cases around the cheilion and in 37 (90.2%) cases around the nasal ala. The cases in which the facial artery was not visible could be explained by an inferior or superior labial pattern.

Previous authors have described clinical and anatomical reference points to identify the facial artery in the perioral region during surgical and cosmetic procedures [22]. However, the tortuous course and the variable branching patterns of the facial artery can limit the reliability of these landmarks. The facial artery runs superficially around the cheilion and the nasal ala, being easily accessible to high-frequency US transducers. Since the accurate identification of the facial artery around the cheilion and in the nasolabial fold is necessary during dermal filler and neurotoxin injection to avoid serious complications such as necrosis of the alar rim, real-time US can be a valuable tool to identify the artery safely.

Moreover, the preoperative identification of the artery in case of a free flap transfer in the upper/midface regions allows to easily access to the recipient's vessels and therefore to reduce the operative time. Although a sonographic evaluation of the more distal branches of the facial artery is technically possible, they have not been included in the current work due to the little consensus about their anatomical variability among the previously published studies [22], the great majority of which were cadaveric and therefore limited in the detection of the smallest and deepest arterial branches not distinguishable with the naked eye [23]. On conventional Doppler US, the small vessels with slow motion velocity can be barely visible due to the wall filters applied to remove clutter and motion artifacts. However, advanced flow-detection imaging techniques, such as the so-called “superb microvascular imaging”, separate flow signals from overlying tissue motion, thus preserving visualization of the subtlest slow-flow vessels [24]. Since these new technologies have overcome the limitations of conventional Doppler imaging, they could be potentially employed in the anatomical study of the distal branches of the facial artery, adding significant details to simple US examination.

While a great variation in the course and branching pattern of the facial artery has been demonstrated, a more constant and predictable course of the facial vein has been described [11, 13, 25]. In our study, US was able to detect the facial vein at all measurement points in all 41 (100%) cases on the right side and in 40 out of 41 (97.6%) cases on the left side. The case in which the left facial vein was not visible could be explained by a transitory vein collapse or by an anatomical variation. On both sides, the median distance of the facial vein from the MO-line did not exceed 2 mm, reflecting an excellent correspondence between the measurement points along the line and the vein course. In an *in vivo* study with computed tomography angiography, Wang et al. [25] regarded the medial canthus, the nasal ala, and the oral commissure as reference points to identify the facial vein. It can be hypothesized that the combined use of these anatomical landmarks and our predictive line can help narrow the area where the vein is most probably localized, assisting in its identification during surgical and injective procedures. Indeed, the retrieval of the facial vein in this particular district during a surgical reconstruction with a free flap is usually difficult and not always possible, and requires significant surgical skills [26, 27].

Following previous studies [15, 16], the facial vein area has been measured, although this parameter has probably been influenced by the subject position during US examination, by the compression exerted by the probe, and by the examination length. Indeed, these are all factors that can alter the venous flow and consequently the vein diameters. Given the contiguity of the facial vein and artery at the inferior border of the mandible, the MO-line can be drawn clinically, after palpation of the adjacent facial artery pulse, or with more precision, after US visualization of the vein at the lower border of the mandible. As for the artery, the facial vein was found to run very superficially on the face, having a maximum mean depth beneath the skin of 10.9 mm at right VP1.

At the nasal ala level (the same level as VP2), Wang et al. [25] found a maximum mean venous depth of 16 mm using computed tomography angiography. This slightly superior value could be due to the different techniques employed since US examination involves a certain degree of soft tissue compression, or to the different sample characteristics, first of which the ethnicity: our study population was Caucasian, while Wang et al. examined only Asian subjects.

At VP3, the mean depth of the facial vein beneath the skin was less than 5 mm, making the vessel detection immediate with last-generation high-frequency US probes. The real-time US assessment of the facial vein could be particularly helpful at VP2 and VP3 levels during dermal injections for tear trough and mid-cheek groove deformities. Furthermore, the three VPs along the MO-line appear as promising landmarks to safely identify the facial vein around the cheilion, the nasal ala, and the orbit. In perspective, the clinical application of the MO-line could help shorten the surgical time during facial flap reconstruction and avoid accidental intravenous injection during cosmetic procedures.

Our study has some limitations. First, the study population is small and ethnically homogenous, therefore larger samples with the inclusion of volunteers of different ethnicity are required to confirm the results. Second, both the predictive-lines draw and the US examination are operator-dependent, so further research should be conducted to assess the intra- and interobserver variability. Third, this is a preliminary observational study and must be followed by a validation study on a larger cohort of patients undergoing surgical or micro-invasive procedures.

In conclusion, high-resolution US is valuable in studying the facial artery and vein and can play an increasingly important role in their real-time assessment prior to surgical procedures. The facial vein has a constant course concerning the MO-line. Therefore, the MO-line seems promising both as a clinical and an imaging-based method to identify the facial vein.

## Data Availability

The datasets used and/or analyzed during the current study are available from the corresponding author on reasonable request.

## Abbreviations

AP1: Arterial point 1
AP2: Arterial point 2
AP3: Arterial point 3
FV: Facial vein
IQR: Interquartile range
MO-line: Mandibular-orbital line
US: Ultrasound
VP1: Venous point 1
VP2: Venous point 2
VP3: Venous point 3
